# Exercise Modality Is Differentially Associated with Neurocognition in Older Adults

**DOI:** 10.1155/2017/3480413

**Published:** 2017-04-18

**Authors:** Yu-Kai Chang, I-Hua Chu, Jen-Hao Liu, Chih-Han Wu, Chien-Heng Chu, Kao-Teng Yang, Ai-Guo Chen

**Affiliations:** ^1^Graduate Institute of Athletics and Coaching Science, National Taiwan Sport University, Taoyuan County, Taiwan; ^2^Department of Sports Medicine, Kaohsiung Medical University, Kaohsiung, Taiwan; ^3^College of Physical Education, Yangzhou University, Jiangsu, China

## Abstract

This study explored the effects of exercise modality and type of fitness index on cognitive function in the older adults as assessed via behavioral and neuroelectrical approaches. Sixty older adults were assigned to an aerobic exercise, a coordination exercise, or a control group based on their previous exercise experience. The participants completed congruent and incongruent trials of a modified Stroop Test, during which, event-related potentials were recorded. The participants also completed multiple physical tests that assessed health- and skill-related fitness. Our findings suggest that, in general, both aerobic and coordination exercise, as well as higher scores on health- and skill-related fitness indices, are positively associated with better performance of various cognitive functions in the elderly population. The mechanisms underlying these relationships may be differentially related to specific neuroelectrical processes involved in neurocognitive control.

## 1. Introduction

Although aging is generally accompanied by the deterioration of multiple facets of cognition [1], extensive research has demonstrated that older adults who regularly engage in physical exercise or who possess a high level of fitness experience a reduced degree of cognitive decline or show improvements in cognitive function. The positive relationships between physical exercise, fitness, and cognitive function have been further demonstrated by a meta-analysis that showed a significant positive effect with the small to large in magnitude [2–4].

The benefits of physical exercise and fitness on cognitive function appear to be disproportionally distributed. Colcombe and Kramer [3] indicated that although exercise training leads to improvements in multiple aspects of cognitive function (i.e., executive function, controlled, spatial, and speed aspects), the executive function aspect of cognition displays the largest enhancement, suggesting that exercise training impacts different types of cognition not only generally but also specifically. Executive function refers to high-level hierarchical cognitive processing that involves inhibitory control, task switching, and working memory [5] to achieve purposeful or goal-directed behavior, particularly in novel situations [6]. The disproportionate improvement in executive function that results from exercise training or fitness in older populations is interesting because executive function is particularly vulnerable to age-related cognitive decline [1]. However, a subsequent meta-analysis indicated that exercise training is moderately associated with cognitive improvements, regardless of whether executive function, attention, processing speed, or memory is considered [7]. This finding demonstrates that the modulatory effects of exercise training on cognitive function remain unclear.

One possibility worth considering is whether exercise modality modulates the relationship between exercise and cognition. The majority of studies have investigated either aerobic exercise (AE) or cardiovascular fitness. Pesce [8] argued the importance of shifting the emphasis from quantitative to qualitative exercise characteristics to advance our understanding of the exercise-cognition relationship. Nonetheless, the effects of other modalities, such as coordination exercise (CE), that involve a variety of exercise characteristics (e.g., muscular strength and endurance, motor coordination, agility, flexibility, and visual-spatial perception) on cognition have received only little or indirect attention until recently. For example, relative to their nonexercising counterparts, older adults in both closed-skill (e.g., jogging) and open-skill (e.g., table tennis) groups demonstrated superior cognitive performances in terms of inhibitory control [9] and task switching [10], which are both aspects of executive function. A similar facilitation of task switching was observed in older adults in both AE and mind-body CE (i.e., Tai Chi Chuan) groups compared with the control [11]. Notably, open-skill exercise and Tai Chi Chuan might be confounded by factors related to environment prediction and light intensity or to the exercise characteristics of meditation. Therefore, the current study replicates and extends existing knowledge by examining the effects of CE that is more closed-skill and more intense than Tai Chi Chuan and that involves less meditation (i.e., routine-based Chinese martial arts).

Cardiovascular fitness has been recognized as a primary behavioral mediator and moderator of AE and cognition. Although cardiovascular fitness is an essential fitness index, it is only one of many fitness indices. Fitness is a multifaceted concept that includes both health-related fitness, involving cardiovascular endurance, muscular strength, muscular endurance, flexibility, body composition, and flexibility, and skill-related fitness, involving agility, power, coordination, balance, reaction time, and speed [12]. Although the relationships between each of these other fitness types and cognition are not yet fully understood [13], a few recent neuroimaging studies showed that both physical fitness (i.e., cardiovascular and muscular strength) and motor fitness indices (i.e., flexibility, motor coordination, movement speed, and balance) not only were positively associated with cognitive performance but also elicited activity in different brain regions [14]. Utilizing cross-sectional and longitudinal approaches, recent neuroimaging studies revealed that both physical and motor fitness are associated with superior and more efficient information processing and enlarged volumes of cognition-related brain areas such as the hippocampus and the basal ganglia [15, 16]. These findings of positive alterations in brain function and structure suggest that types of fitness other than cardiovascular fitness may be positively associated with cognitive performance. However, these studies categorized two types of fitness indices within the category of physical fitness and categorized health- and skill-related fitness indices within the category of motor fitness. As such, the relationships between specific fitness indices and cognition have yet to be determined.

Neuroelectrical studies using event-related potentials (ERPs) have provided insight beyond overt behavioral responses into the potential mechanisms underlying the relationships among exercise, fitness, and cognition [17, 18]. ERPs are characterized by high temporal sensitivity between stimulus engagement and response execution and are believed to reflect implicit and distinct cognitive processes that are reflected by specific ERP components. For example, P3, a positively deflecting waveform that occurs between 300 and 600 ms following stimulus onset, represents the amount of attentional resources allocated to a given task [19]. Previous ERP studies have revealed positive relationships between exercise and neurocognitive performance in executive function-related tasks; older adults with a higher fitness status or who are more engaged in physical activity demonstrate greater P3 amplitudes than those with a lower fitness status or who are less engaged in physical activity, respectively [17, 18, 20, 21]. Similarly, larger P3 amplitudes were recently observed in older adults engaged in either AE or CE compared with controls, with no difference in P3 amplitude between these two modalities [10, 11]. These results suggest that exercise enhances neuroelectrical activation that is related to higher cognitive performance regardless of the exercise modality and fitness type. Although P3 has been extensively examined, the utilization of executive tasks and the study of exercise and other ERP components have been limited. The current study utilized the Stroop Test, a widely used and recommended neuropsychological assessment of executive function [6, 22]. In the Stroop Test, subjects in the incongruent condition, in which the name of the color is different from the meaning of the word, show increased reaction times and decreased accuracy compared with their performance under the congruent condition, in which the name of the color is the same as the meaning of the word. These behavioral differences are believed to be associated with the inhibition aspect of executive function [22, 23]. The combination of the ERP paradigm with the Stroop Test not only incorporates congruent and incongruent trials that reflect information processing and executive function, respectively, but also elicits late (i.e., P3 and N450) and early components (i.e., N1 and N2) [24], facilitating the simultaneous evaluation of the nature of cognitive function and multiple ERP components.

Accordingly, the purposes of the current study were to investigate (a) whether AE and CE are generally or specifically associated with multiple cognitive functions as assessed by the Stroop Test, (b) the correlations between cognitive functions and cardiovascular fitness versus other health-related (i.e., muscular strength and endurance, body composition, and flexibility) and skill-related fitness measures (i.e., agility and power), and (c) the mechanisms underlying the effects of AE and CE on executive-function task performance based on the time course of early and late neuroelectrical activation. It was predicted that individuals engaged in exercise of two modalities would demonstrate superior cognitive performance, both generally and specifically. It was also predicted that fitness, regardless of type, would correlate positively with cognitive performance. Finally, we predicted that exercise of both modalities would not only induce a larger P3 amplitude during a cognitive task but would also affect other ERP components that reflect different aspects of neuroelectrical processing.

## 2. Materials and Methods

### 2.1. Participants

Healthy older adults were recruited via flyers posted in universities, communities, hospitals, parks, jogging clubs, and Chinese Martial Art/Kung Fu establishments (clubs for routine-based Chinese martial arts that involve a series of complex and intense motor skills). Participant referrals were also received from the greater New Taipei and Taipei regions of Taiwan. Eligible participants met the following initial criteria: (a) age between 55 and 70 years, (b) score ≥ 26 on the Mini-Mental State Exam (MMSE), (c) no psychiatric or neurological disorders, (d) no history of stroke or head injury, (e) normal or corrected-to-normal vision without color blindness, (f) right hand dominance, and (g) score ≥ 7 on the Physical Activity Readiness Questionnaire (PAR-Q).

The participants in the AE and CE groups were required to meet additional criteria based on a self-reported exercise experience survey. The AE and CE groups included older adults who regularly participated in AE (i.e., jogging, walking, and/or swimming) and CE (i.e., Chinese Marital Art and/or Tai Chi Chuan), respectively, for at least 30 minutes per session and three times per week for the previous 6 months. The control group included older adults who irregularly participated in exercise (i.e., less than two times per week). Each group included 20 participants, with a total of 60 participants. The sample size was sufficiently sensitive to reveal differences in cognitive function according to the G∗Power 3 method based on Colcombe and Kramer [3]. All of the participants read and signed an informed consent form that was approved by the Institutional Review Board of National Taiwan University.  Table 1 presents the participant demographic, exercise experience, and fitness data.

### 2.2. Assessment of Health-Related Fitness

#### 2.2.1. Cardiorespiratory Fitness

Cardiovascular fitness was evaluated based on estimated peak oxygen consumption (VO_2peak_) using the submaximal exercise test of the YMCA cycle ergometry protocol [25]. This protocol, recommended for individuals of a Class A risk stratification [26], consisted of two to four three-minute stages with a progressively increasing workload. VO_2peak_ was estimated based on the slope of the heart rate, the workload, and the body weight. During testing, objective and subjective assessments of exercise intensity were conducted using a Polar heart rate monitor (Sport Tester PE 3000, Polar Electro Oy, Kempele, Finland) and a 6- to 20-point version of the rating of perceived exertion (RPE) scale, respectively [27].

#### 2.2.2. Muscular Fitness

Muscular strength was defined as the average hand force assessed with a handgrip dynamometer (three attempts each for the left and the right hand). Muscular endurance was defined as the number of push-ups (regular push-ups for males and knee push-ups for females) in one minute and the number of abdominal crunches/curl-ups in 30 seconds for males and 60 seconds for females.

#### 2.2.3. Flexibility and Body Composition

Flexibility was assessed using the YMCA sit-and-reach test and was reported relative to the distance (cm) between the hamstring and the lower back. Body composition was presented as the body mass index and was measured by bioimpedance spectroscopy (InBody 3.0 DS12B887, Dallas, TX, USA) by determining the percentage of body fat mass.

### 2.3. Assessment of Skill-Related Fitness

The skill-related fitness measures agility and power were evaluated as the time to complete the T-test and the distance of the vertical jump test, respectively [28]. During the T-test, each participant was asked to jump to different corners arranged as T-figures. During the vertical jump test, participants were asked to jump from a static position as fast and as high as possible.

### 2.4. Stroop Test

This task was modified from the Stroop Color-Word task [29] and consisted of 6 blocks of 60 trials. The task involved two types of trials: congruent and incongruent. In the congruent trials, one of three Chinese color words (紅RED, 綠 GREEN, and 藍 BLUE) was presented, printed in the same color ink (e.g., RED printed in red ink). Incongruent trials consisted of one of the same three words printed in a different ink color (e.g., RED printed in green ink). One-third of the trials were incongruent, and the remaining trials were congruent; the trials were randomly presented. In each trial, a fixation cross (+) appeared in the center of the screen for 506 ms and followed by stimulus presentation for 500 ms. The participants were instructed to respond based on the color of the ink and to ignore the meaning of the word by pressing one of the three colored buttons on a response pad. The button colors corresponded to ink colors. The response was considered as incorrect if no response was recorded within 1000 ms following stimulus presentation. The stimuli were 2 cm^2^ in size and were presented in the center of a 15-inch screen, with a visual angle of 28.14 × 1.4°. All of the participants performed 20 practice trials prior to beginning the official task. The reaction times and the accuracy of each participant were recorded and analyzed as the behavioral outcome measures.

### 2.5. EEG Recording and ERP Measures

All participants were instructed to sit in a comfortable chair in an electrically shielded electroencephalography (EEG) recording chamber with attenuated sound levels. The participants focused on the center of the screen and made minimal body movements during the recording. The EEG data were recorded from 32 Ag/AgCl electrodes embedded in an elastic cap (Quick-Cap, NeuroScan Inc.) and positioned in accordance with the standard 10–20 system [30]. During recording, the impedance of all electrodes was maintained at or below 10 kΩ. Online EEG recording data were referenced to the left and right mastoids, and the AFz electrode site served as the ground. The EEG data were sampled at 1000 Hz, filtered using an online band-bass filter (0.05–70 Hz), and DC-amplified. A 60 Hz notch filter was applied using a SynAmps^2^ amplifier system. Electrooculographic (EOG) activity was recorded using two additional sets of electrodes, which were located at the outer canthus of each orbit and above and below the left orbit. These sets of electrodes recorded the horizontal and vertical electrooculograms.

The offline EEG data were corrected for ocular artifacts using the eye movement correction algorithm of the NeuroScan software. The stimulus-locked epochs acquired for the Stroop trials were extracted offline from 200 ms prestimulus onset to 1000 ms poststimulus onset, and the period from 100 to 0 ms prestimulus onset was used as the baseline. The data were filtered using a zero phase shift, 30 Hz (12 dB/oct) low-pass filter. Trials were rejected if the response was incorrect or if the voltage exceeded ±100 *μ*V. Following the offline analysis processes, ERP data from six participants were excluded (two from the control group and four from the CE group). The grand average waveform across all accepted trials was calculated. The poststimulus onset time windows used for the calculation of the mean amplitude of each ERP component at Fz, Cz, and Pz were 80–150 ms for N1, 200–300 ms for N2, 350–550 ms for P3, and 400–500 ms for N450. The topographic distribution of the specific components across all of the electrode sites is presented.

### 2.6. Procedure

The participants were required to come to the laboratory of National Taiwan Sport University. Eligibility was assessed using a demographic questionnaire, a health-screening questionnaire, the MMSE, the PAR-Q, and a survey about exercise experience. The eligible participants were administered with the digit-span test of the Wechsler Adult Intelligence Scale-Third Edition (WAIS-III) to assess the working memory component of intelligence [31] and were instructed to conduct the Stroop Test during EEG recording. Following the completion of cognition testing, the electrodes were removed, and body composition, muscular-related fitness, and flexibility were measured by trained experimenters, followed by measurements of agility, power, and cardiorespiratory fitness. The experimental procedure spanned approximately two hours. The participants were informed of the purpose of the study and received $30 in remuneration.

### 2.7. Statistical Analysis

The characteristics of the participants were compared among the three groups using a one-way analysis of variance (ANOVA). The behavioral measures of reaction time and accuracy were assessed separately via a 3 (Group: control, CE, AE) × 2 (Stroop congruency: congruent, incongruent) repeated-measures ANOVA. Pearson product-moment correlations were used to examine the associations between the fitness variables and the behavioral measures. The neuroelectrical measures for each ERP component (i.e., N1, N2, P3, and N450) were analyzed separately using a 3 (Group) × 2 (Stroop congruency) × 3 (Site: Fz, Cz, and Pz) repeated-measures ANOVA. Multiple pairwise comparisons were Bonferroni-corrected and applied for post hoc comparison. Statistical values were presented following Greenhouse-Geisser corrections, in which a partial eta-square (*η*_*p*_^2^) value was provided for the significant effects. The family-wise alpha value of 0.05 was set prior to Bonferroni adjustment.

## 3. Results

### 3.1. Participant Characteristics

One-way ANOVA revealed significant differences among groups only for the fitness-related variables (Table 1, *p* < 0.001). Post hoc comparison indicated that the VO_2peak_ value of the AE group was significantly higher than the values of the CE and control groups and that the VO_2peak_ value of the CE group was significantly higher than that of the control group. Muscular strength, endurance, flexibility, and power were significantly higher in both the AE and CE groups than in the control group, whereas no differences were observed between the AE and CE groups. In addition, higher % body fat mass was observed in the control group than those in the AE and CE groups. Regarding agility, the AE and CE groups were significantly faster than the control group, with no difference observed between the AE and CE groups.

### 3.2. Behavioral Measures

Table 2 summarizes the behavioral and neuroelectrical values (mean and SE) for the two congruency conditions across the three groups.

#### 3.2.1. Reaction Time

**Figure 1:**
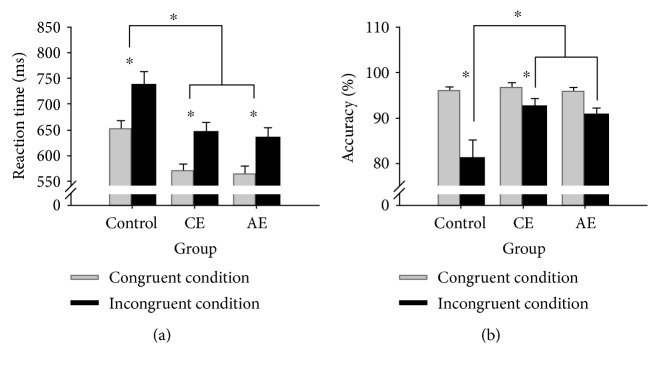
A comparison of the behavioral measures according to Stroop congruency for the three groups: (a) reaction time; (b) accuracy. The data are presented as the means ± SEM. ^∗^*p* < 0.05. CE = coordination exercise; AE = aerobic exercise.

Two-way ANOVA revealed a main effect of Group (*F*_2,57_ = 9.82, *p* < 0.001, *η*_*p*_^2^ = 0.26), with a shorter reaction time for each of the AE and CE groups than that for the control group (*p* < 0.002 for both), and a main effect of Stroop congruency (*F*_1,57_ = 258.42, *p* < 0.001, *η*_*p*_^2^ = 0.82), with a longer reaction time under the incongruent condition than that under the congruent condition (*p* < 0.001) (Figure 1(a)).

#### 3.2.2. Accuracy

Two-way ANOVA revealed a main effect of Group (*F*_2,57_ = 4.06, *p* < 0.02, *η*_*p*_^2^ = 0.16) and a main effect of Stroop congruency (*F*_1,57_ = 43.33, *p* < 0.001, *η*_*p*_^2^ = 0.43) that was superseded by a Group × Stroop congruency interaction (*F*_2,57_ = 8.25, *p* < 0.001, *η*_*p*_^2^ = 0.23). Post hoc comparison indicated higher accuracy in the AE and CE groups than that in the control group under the incongruent condition (*p* < 0.02 for both), whereas no difference in accuracy was found among the three groups under the congruent condition. In addition, higher accuracy was observed under the congruent condition than under the incongruent condition in the control and CE groups (*p* < 0.02 for all), but not in the AE group (Figure 1(b)).

#### 3.2.3. Correlation Analysis

Table 3 summarizes the Pearson product-moment correlations between the fitness variables and the behavioral measures. Generally, VO_2peak_, muscular strength, muscular endurance, and power were negatively correlated with reaction time under both the congruent and incongruent conditions (*p* < 0.02 for all). Agility and body composition were positively correlated with reaction time under both the congruent and incongruent conditions (*p* < 0.004 for all). However, no significant relationship between flexibility and reaction time was observed.

### 3.3. ERP Measures

**Figure 2:**
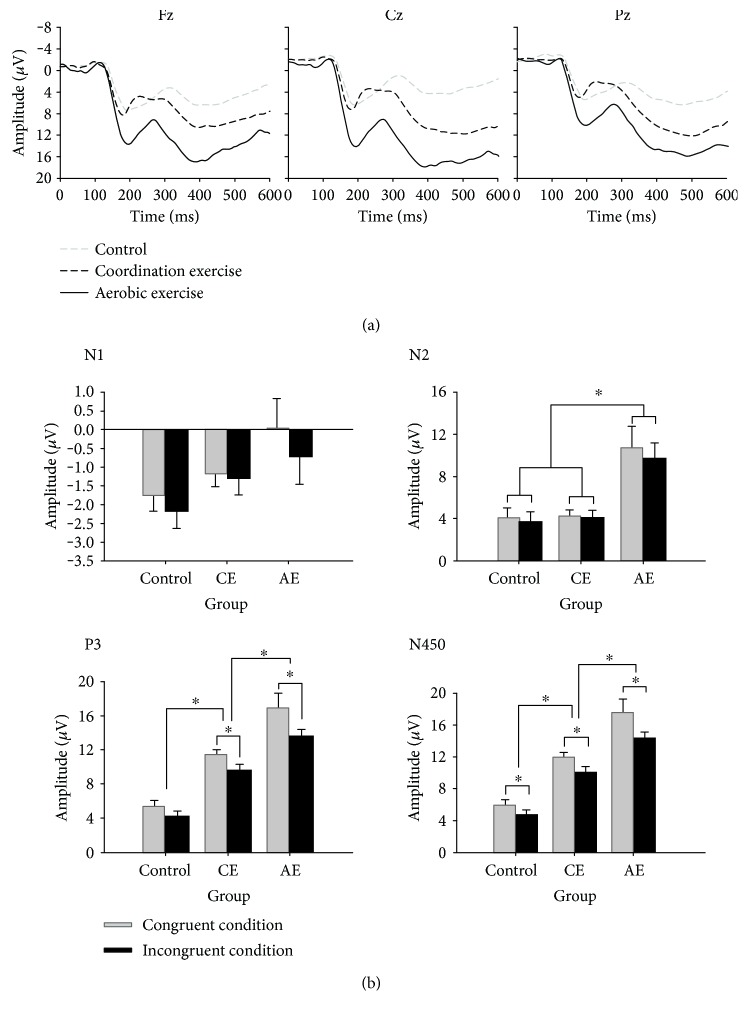
(a) Grand average ERP waveform of averaged Stroop congruency in each group at the Fz, Cz, and Pz electrode sites. (b) Interaction effect between Stroop congruency and group for each ERP component on N, N2, P3, and N450. CE = coordination exercise; AE = aerobic exercise. ^∗^*p* < 0.05.

**Figure 3:**
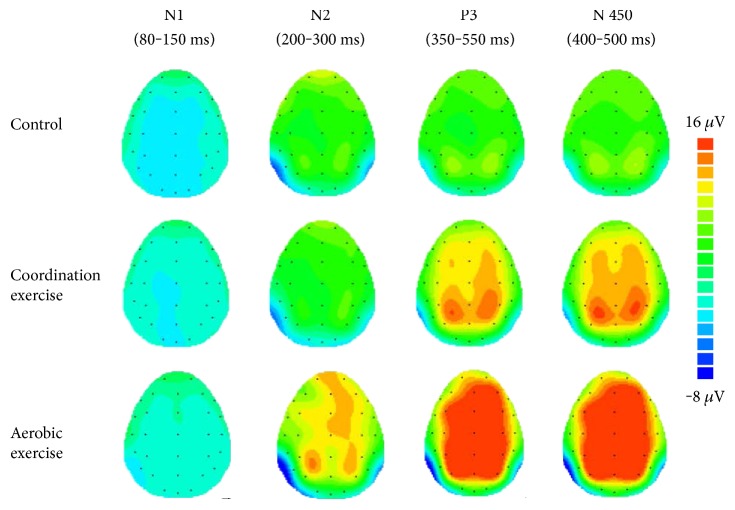
Topographic distribution of each ERP component (i.e., N1, N2, P3, and N450) across the global scalp for the three groups.

Figure 2 illustrates the grand average ERP waveform of each Stroop congruency for each group and the interaction between Stroop congruency and Group.  Figure 3 illustrates the topographical distribution of each ERP component (i.e., N1, N2, P3, and N450) across the global scalp for the three groups.

#### 3.3.1. Mean N1 Amplitude

Three-way ANOVA revealed a main effect of Site (*F*_2,102_ = 32.98, *p* < 0.001, *η*_*p*_^2^ = 0.39), which was superseded by a Site × Stroop congruency × Group interaction (*F*_4,57_ = 3.55, *p* < 0.02, *η*_*p*_^2^ = 0.12). Post hoc comparisons indicated greater N1 amplitude for Cz and Pz than that for Fz in the three groups (*p* < 0.008 for all) and under both congruency conditions (*p* < 0.001 for all). No other significant main effects or interactions were revealed.

#### 3.3.2. Mean N2 Amplitude

Three-way ANOVA revealed main effects of Group (*F*_2,51_ = 9.03, *p* < 0.001, *η*_*p*_^2^ = 0.26) and Site (*F*_2,102_ = 23.83, *p* < 0.001, *η_p_*^2^ = 0.32), which were superseded by a Group × Stroop congruency interaction (*F*_4,102_ = 4.20, *p* < 0.007, *η*_*p*_^2^ = 0.14). Post hoc comparisons indicated a smaller N2 amplitude in the AE group than in the CE and control groups under both congruency conditions (*p* < 0.004 for both). No other significant main effects or interactions were revealed.

#### 3.3.3. Mean P3 Amplitude

Three-way ANOVA revealed main effects of Group (*F*_2,51_ = 39.16, *p* < 0.001, *η*_*p*_^2^ = 0.61) and Stroop congruency (*F*_1,51_ = 18.89, *p* < 0.001, *η*_*p*_^2^ = 0.27), which were superseded by a Group × Stroop congruency interaction (*F*_4,102_ = 7.89, *p* < 0.001, *η*_*p*_^2^ = 0.24). Post hoc comparisons indicated that the greatest P3 amplitude was observed in the AE group, followed by the CE group (*p* < 0.005) and the control group (*p* < 0.005), under both congruency conditions. However, a difference in P3 amplitude between the congruent and incongruent conditions was only observed in the AE and CE groups (*p* < 0.05 for both), not in the control group. No other significant main effects or interactions were revealed.

#### 3.3.4. Mean N450 Amplitude

Three-way ANOVA revealed a main effect of Group (*F*_2,51_ = 37.11, *p* < 0.001, *η*_*p*_^2^ = 0.59), which was superseded by a Group × Site interaction (*F*_4,102_ = 7.16, *p* < 0.001, *η*_*p*_^2^ = 0.22). Post hoc comparisons indicated that the smallest N450 amplitude was observed in the AE group, followed by the CE group (*p* < 0.02 for all) and the control group (*p* < 0.02 for all), for all three sites. In addition, although no differences in N450 amplitude were found among the three sites in the CE group, Fz and Pz displayed higher N450 amplitudes than did Cz in the AE and control groups. The analysis also revealed a main effect of Stroop congruency (*F*_1,51_ = 22.70, *p* < 0.001, *η*_*p*_^2^ = 0.31), with a greater N450 amplitude under the incongruent condition than under the congruent condition (*p* < 0.001). No other significant main effects or interactions were revealed.

## 4. Discussion

The current study, which investigated exercise-cognition relationships in older adults, is among the first to examine the modulatory role of exercise modality on cognitive function, as assessed by the Stroop Test, from behavioral and neuroelectrical perspectives. The major finding was that the AE and CE groups both demonstrated shorter reaction times than those of the control group in both the congruent and incongruent trials. In addition, higher ratings on all the health- and skill-related fitness indices except flexibility were positively associated with cognitive performances, and positive relationships between fitness and cognition were observed regardless of the type of cognitive function assessed. Examination of the time course of the ERP components indicated that the AE group exhibited the largest P3 amplitude and the smallest N2 and N450 amplitudes compared with the corresponding amplitudes of the other two groups. The CE group exhibited a larger P3 amplitude and a smaller N450, but not N2, amplitude than did the control group. However, no difference in N1 amplitude was observed among the three groups.

Our first aim was to test whether exercise modality was associated with cognition either generally or specifically. The prolonged reaction times and reduced accuracies observed in the incongruent trials suggest that these trials require greater cognitive demand than do the congruent trials, possibly due to interference. The observed differences between the congruent and incongruent trials not only represent the typical “Stroop interference effect” but also demonstrate the appropriateness of our task manipulation. Additionally, along with the finding of an increase or no change in accuracy between each exercise group and the control group, the association of superior cognitive performance with exercise in both types of congruency trials excludes the possibility of a speed-accuracy tradeoff. Although these results contrast to some degree with the specific improvement hypothesis, our findings agree with empirical studies that showed similar improvements in performance on the flanker task for congruent and incongruent trials [20] and on working memory task with different loads [21], suggesting a general improvement associated with AE.

However, a novel finding of the present study was that CE elicited similar positive effects as AE on the behavioral measures. That is, general improvements in behavioral cognitive performance were exhibited by older adults in the CE group as well as those in the AE group. These findings from the Stroop Test extend the findings of previous studies that focused on the effect of CE on performance on the flanker task [14, 32] and the effects of open-skill exercise and light-intensity CE on performance on a task-switching test [10, 21]. These results might be particularly important because Stroop Test performance has been revealed to decline with age [33]. CE that involves a variety of exercise characteristics may enable environmental enrichment to increase cognitive performance. An extensive rodent study has shown that environmental enrichment consisting of repeated exercise, complex motor skills, cognitive stimulation, and/or social interaction has a positive effect on neurogenesis, neurotrophin expression, and synaptic plasticity involved in memory and learning [34]. Furthermore, an enriched environment leads to more cell proliferation than running exercise alone [34], suggesting an association between complex exercise modalities and cognition. Taken together, these findings suggest that the positive associations between exercise and cognition in older adults are independent of cognitive function type, executive function type, and exercise modality.

Although both exercise groups demonstrated better fitness index values than the control group, the AE and CE groups exhibited higher cardiovascular fitness and greater flexibility, respectively. These findings suggest that the relationship between exercise modality and cognition may be interpreted from the perspective of fitness type [16, 32], which was our second aim. Our findings demonstrated that cognitive function, as assessed by the Stroop Test, was positively correlated with each of the cardiovascular, muscular (i.e., strength and muscular endurance), body composition, and agility fitness indices. Thus, our study provides the first demonstration that, with the exception of flexibility, fitness is associated with improved cognitive performance regardless of whether the measures are health- or skill-related fitness indices. Although both types of fitness demonstrated similar positive effects on cognition, the brain plasticity associated with the fitness indices and cognition might differ. For example, greater hippocampal volume was observed in older adults with high cardiovascular fitness [35] or high motor fitness [16] compared with the volume in their less-fit counterparts. In contrast, the volume of basal ganglia, which is responsible for the early stages of motor learning and the processes of executive function, was positively associated only with motor fitness and mediated the association between motor fitness and executive function [16]. Similarly, physical fitness (i.e., cardiovascular and muscular fitness) is primarily related to the activation of sensorimotor cortical areas, whereas motor fitness indices are primarily related to the activation of visuo-motor and visuo-spatial networks [14, 32]. In contrast, older adults who follow long-term resistance exercise interventions demonstrate better cognitive performance but display smaller frontal and temporal brain volumes [36]. These findings suggest differential neuromodulation with respect to the type of fitness. Future study is warranted, including the simultaneous examination of health- and skill-related fitness and cognitive functions using a neuroimaging approach.

Another novel aspect of the current study was the monitoring of the time course of ERPs, including both late (i.e., P3 and N450) and early components (i.e., N1 and N2). Our P3 findings replicate and extend previous results showing a positive association between P3 and exercise [17, 18, 20, 21]. In the present study, older adults in the AE group exhibited a larger P3 amplitude than those in the control group. Notably, the P3 amplitudes of the CE group were also larger than those of the control group but were smaller than those of the AE group. This result is in accordance with studies that examined either open-skill exercise or Tai Chi Chuan [10, 11]. These findings suggest that in addition to improving cognitive performance, participation in exercise, regardless of the exercise modality, induces an increase in attentional resource allocation during cognitive processing.

Interestingly, N450, another late component, showed a similar pattern to that of P3. The AE group had the smallest N450 amplitudes, followed by the CE group, and, finally, the control group. N450, a specific component induced by the Stroop Test, is believed to originate in the anterior cingulate cortex (ACC) and to reflect conflict detection processes [37]. Our finding of greater N450 amplitudes following incongruent trials than following congruent trials support evidence of a role of N450 in conflict monitoring activity [38]. The association between N450 activity and the ACC provides an alternative explanation for the beneficial effect of exercise. Colcombe et al. indicated that both older adults with higher cardiovascular fitness and those performing AE training exhibit better executive function, with lower activation in the ACC [39]. Accordingly, from a neuroelectrical perspective, our finding of a decrease in N450 amplitude due to exercise is consistent with the superior performance of the AE and CE groups on the Stroop Test because of the reduction of ACC activation, extending these findings from AE to CE.

The early ERP components displayed largely distinct activity from that of the late ERP components. Specifically, a reduced N2 amplitude was observed only in the AE group, and no differences in N1 were observed among the three groups. N2 amplitude is susceptible to the degree of conflict and is believed to be associated with conflict monitoring by the ACC [40]. Previous studies that investigated the association between N2 and fitness have predominately focused on younger or older children, and our findings demonstrate that the association of reduced N2 amplitude with AE extends to older adults [41, 42]. These N2 findings, along with the reduction in the N450 amplitude in the AE group, suggest that AE is positively associated with improved top-down executive control and reduced conflict processing. However, CE failed to show this N2 alteration, and this result is inconsistent with our prior expectations. Despite their similar origin in the ACC and reflection of conflict processing, N2 and N450 may be distinguishable. N2 is believed to represent conflict detection, adjustment, and resolution [24, 40], whereas N450 reflects only conflict detection within conflict monitoring processes [40]. Our data reflect that the effects of each exercise modality may be modulated by the relationship between exercise and conflict monitoring; specifically, AE likely affects a variety of subprocesses within conflict monitoring, and CE specifically impacts conflict detection. Future research is required to replicate these findings and to test this hypothesis.

The current study failed to identify a difference in N1 among the three groups. N1 is an exogenous ERP component that reflects stimulus discrimination and is primarily controlled by the physical characteristics of the event. To date, only a few studies have examined N1. Chang et al. [21] reported that N1 amplitudes were larger in older adults with more physical activity than in those with less physical activity, conflicting with our findings. However, their study utilized a working memory task in which the memory response of the retrieval phase depended on the stimulus in the preceding encoding phase, whereas the cognitive inhibition of the Stroop Test utilized in the current study might require less effort at the early stage of information processing. Therefore, the lack of an observed association of alteration in N1 activity with exercise in the present study may indicate that the effect of exercise on effortful stimulus discrimination at the early stage of the Stroop Test is limited.

Although the present study extends current knowledge by examining the relationships among exercise, fitness, and cognitive function using behavioral and neuroelectrical approaches, caution must be taken when interpreting the findings. The positive associations among exercise modalities, fitness indices, and cognitive function observed in the current study were based on cross-sectional evidence, which limits the interpretation of causal relationships. However, along with recent neuroimaging studies that used longitudinal interventions to show that exercise leads to modifications of brain function and structure [15, 16], our findings indicate that further exploration of the effects of different exercise modalities and fitness measures on cognitive function is warranted, using randomized controlled trials and neuroelectrical approaches. Although the older adults in the CE group exhibited lower cardiovascular fitness and greater flexibility than those in the AE group, the two groups exhibited similar ratings on the other health- and skill-related fitness indices. To determine the effectiveness of an exercise modality and the importance of a specific fitness index, exercise designed to involve high muscular demand in addition to intense motor and coordination demand is required in older adults. A potential confounding factor in the present study is the gender ratio, which differed among the groups. Although we found no significant gender differences regarding the three measures of the WAIS-III, our findings should be interpreted with caution. Finally, although the emphasis on inhibitory control and the selection of the Stroop Test were appropriate, exercise might differentially affect specific aspects of executive function (e.g., working memory and task switching), or its effects may depend on the neuropsychological assessment utilized [6]. Thus, further investigation of the exercise-cognition relationship by comparing different types of executive function using a variety of tasks is suggested.

## 5. Conclusions

Previous studies examined the relationships among AE, cardiovascular fitness, and cognition. Our study extended this knowledge based on demonstrating the effectiveness of AE and CE for improving cognitive performance, irrespective of the aspect of cognitive function, as indicated by the results of the Stroop Test. Although we provided the first demonstration that the positive association between fitness and cognition is independent of the measure of health- or skill-related fitness assessed, different exercise modalities may differentially impact neuroelectrical activation. Specifically, both AE and CE induced enhanced allocation of attentional resources and improved conflict detection during conflict monitoring processes, although AE appeared to have an additional effect on conflict adjustment and resolution. In addition to clarifying the association between fitness and cognition and providing the potential mechanism underlying this relationship, the present research provides an important implication to serve as a reference for the selection of exercise modalities to improve cognitive function.

## Figures and Tables

**Table 1:** Participant demographic and fitness data for the three groups (mean ± SD).

Measures	Group		
	Control (*n* = 20)	Coordination (*n* = 20)	Aerobic (*n* = 20)
Female/male	5/15	7/13	10/10
Age (year)	57.51 ± 3.68	59.15 ± 4.62	58.80 ± 3.82
Height (cm)	159.13 ± 0.15	162.52 ± 0.31	161.55 ± 0.01
Weight (kg)	67.1 ± 9.52	63.62 ± 9.77	61.22 ± 6.84
Education	9.30 ± 3.62	10.01 ± 0.12	9.52 ± 2.12
MMSE	27.71 ± 2.31	27.82 ± 1.01	28.23 ± 2.60
Resting HR	66.30 ± 11.00	67.41 ± 12.14	65.25 ± 5.29
WAIS-III			
Digit forward span test	12.00 ± 2.43	12.45 ± 1.82	12.55 ± 2.35
Digit backward span test	6.30 ± 1.71	6.40 ± 1.86	6.30 ± 2.83
Digit span total	17.50 ± 3.50	18.85 ± 2.30	19.15 ± 4.68
Exercise characteristics			
Exercise years	0.10 ± 0.31	3.80 ± 1.47	3.20 ± 1.24
Exercise duration/session	0.11 ± 0.00	1.80 ± 0.41	1.85 ± 0.86
Session/week	0.01 ± 0.00	1.15 ± 0.81	1.85 ± 0.75
Fitness data			
VO_2peak_ (mL/kg min)	26.91 ± 6.14	33.67 ± 8.24	41.16 ± 9.73
Muscular strength	51.45 ± 17.72	71.40 ± 20.01	71.65 ± 20.49
Muscular endur./press-up	2.50 ± 3.71	11.40 ± 10.81	12.70 ± 9.80
Muscular endur./CCU 30	4.10 ± 4.18	11.25 ± 5.025	12.95 ± 6.35
Muscular endur./CCU 60	5.70 ± 5.76	19.60 ± 8.55	20.35 ± 11.63
Flexibility (cm)	24.90 ± 11.43	37.20 ± 7.58	35.60 ± 9.51
% body fat mass	30.17 ± 6.58	25.41 ± 5.47	24.97 ± 4.60
Agility (sec)	25.10 ± 6.05	18.12 ± 2.85	18.24 ± 2.67
Power (cm)	21.65 ± 7.85	31.95 ± 7.38	33.35 ± 12.56

MMSE: Mini-Mental State Exam; Muscular endur.: muscular endurance; WAIS-III: Wechsler adult intelligence scale-third edition.

**Table 2:** Behavioral and neuroelectrical data for the two congruency conditions in the three groups (mean ± SEM).

Measures	Group		
	Control	Coordination	Aerobic
Reaction time (ms)			
Congruent trial	651.69 ± 14.24	570.89 ± 14.24	565.01 ± 14.24
Incongruent trial	738.89 ± 19.78	647.43 ± 19.78	636.31 ± 19.78
Accuracy (%)			
Congruent trial	0.96 ± 0.01	0.97 ± 0.01	0.96 ± 0.01
Incongruent trial	0.81 ± 0.02	0.93 ± 0.02	0.91 ± 0.02
N1 amplitude (*μ*V)			
Congruent trial	−1.76 ± 059	−1.18 ± 0.63	0.03 ± 0.56
Incongruent trial	−2.18 ± 058	−1.31 ± 0.61	−0.73 ± 0.55
N2 amplitude (*μ*V)			
Congruent trial	4.13 ± 1.4	4.23 ± 1.49	10.76 ± 1.33
Incongruent trial	3.77 ± 1.1	4.16 ± 1.16	9.74 ± 1.04
P3 amplitude (*μ*V)			
Congruent trial	5.52 ± 1.23	11.45 ± 1.3	17.39 ± 1.16
Incongruent trial	4.42 ± 0.62	9.69 ± 0.66	14.03 ± 0.59
N450 amplitude (*μ*V)			
Congruent trial	5.94 ± 1.2	11.92 ± 1.27	17.54 ± 1.14
Incongruent trial	4.82 ± 0.64	10.07 ± 0.68	14.35 ± 0.61

**Table 3:** Pearson product-moment correlation matrix for the fitness variables and the behavioral measures.

Measures	1	2	3	4	5	6	7	8	9
(1) VO_2max_	1								
(2) Muscular strength (+)	0.48^∗∗^	1							
(3) Muscular endur./press-up (+)	0.47^∗∗^	0.58^∗∗^	1						
(4) Muscular endur./CCU 30 (+)	0.41^∗∗^	0.65^∗∗^	0.64^∗∗^	1					
(5) Muscular endur./CCU 60 (+)	0.44^∗∗^	0.61^∗∗^	0.6^∗∗^	0.96^∗∗^	1				
(6) Flexibility (+)	0.22^∗^	0.25^∗^	0.29^∗^	0.32^∗∗^	0.3^∗^	1			
(7) Agility (−)	−0.48^∗∗^	−0.43^∗∗^	−0.49^∗∗^	−0.62^∗∗^	−0.62^∗∗^	−0.4^∗∗^	1		
(8) Power (+)	0.62^∗∗^	0.81^∗∗^	0.59^∗∗^	0.7^∗∗^	0.72^∗∗^	0.2	−0.6^∗∗^	1	
(9) Body composition (−)	−0.53^∗∗^	−0.57^∗∗^	−0.47^∗∗^	−0.41^∗∗^	−0.42^∗∗^	0.03	0.49^∗∗^	−0.72^∗∗^	1
(10) Accuracy (%) Cong. (+)	0.16	0.04	−0.09	−0.14	−0.15	−0.04	−0.14	0.08	−0.23^∗^
(11) Accuracy (%) Incong. (+)	0.29^∗^	0.21	0.25^∗^	0.1	0.15	0.18	−0.28^∗^	0.28^∗^	−0.34^∗∗^
(12) Reaction time (ms) Cong. (−)	−0.27^∗^	−0.32^∗∗^	−0.45^∗∗^	−0.36^∗∗^	−0.39^∗∗^	−0.16	0.37^∗∗^	−0.39^∗∗^	0.42^∗∗^
(13) Reaction time (ms) Incong. (−)	−0.24^∗^	−0.27^∗^	−0.41^∗∗^	−0.31^∗∗^	−0.36^∗∗^	−0.09	0.34^∗∗^	−0.35^∗∗^	0.34^∗∗^

Cong.: congruent condition; Incong.: incongruent condition. ^∗^*p* < 0.05; ^∗∗^*p* < 0.01.
